# Lateral Flow Assay for the Detection of African Swine Fever Virus Antibodies Using Gold Nanoparticle-Labeled Acid-Treated p72

**DOI:** 10.3389/fchem.2021.804981

**Published:** 2022-01-03

**Authors:** Wenzhuang Zhu, Kaiwen Meng, Yueping Zhang, Zhigao Bu, Dongming Zhao, Geng Meng

**Affiliations:** ^1^ College of Veterinary Medicine, China Agricultural University, Beijing, China; ^2^ State Key Laboratory of Veterinary Biotechnology, National High Containment Facilities for Animal Diseases Control and Prevention, Harbin Veterinary Research Institute, Chinese Academy of Agricultural Sciences, Harbin, China

**Keywords:** African swine fever virus, gold nanoparticle, lateral flow assay, p72, acid treatment

## Abstract

African swine fever is a widespread and highly contagious disease in the porcine population, which is caused by African swine fever virus (ASFV). The PCR and ELISA detection methods are the main conventional diagnostic methods for ASFV antigen/antibody detection in the field. However, these methods have limitations of expensive equipment, trained technicians, and time-consuming results. Thus, a rapid, inexpensive, accurate and on-site detection method is urgently needed. Here we describe a double-antigen-sandwich lateral-flow assay based on gold nanoparticle-conjugated ASFV major capsid protein p72, which can detect ASFV antibody in serum samples with high sensitivity and specificity in 10 min and the results can be determined by naked eyes. A lateral flow assay was established by using yeast-expressed and acid-treated ASFV p72 conjugated with gold nanoparticles, which are synthesized by seeding method. A high coincidence (97.8%) of the assay was determined using clinical serum compared to a commercial ELISA kit. In addition, our lateral flow strip can detect as far as 1:10,000 diluted clinically positive serum for demonstration of high sensitivity. In summary, the assay developed here was shown to be rapid, inexpensive, accurate and highly selective. It represents a reliable method for on-site ASFV antibody detection and may help to control the ASFV pandemic.

## Introduction

Beginning in the summer of 2018, African swine fever virus (ASFV) emerged worldwide and has not been properly controlled thus far (Zhou et al., 2018). It is a highly contagious and lethal hemorrhagic disease in domestic pigs that has spread through many Caucasus countries, the Russian Federation, and Eastern Europe over the past decade (Anderson et al., 1998; J. et al., 1969; Sánchez-Cordón et al., 2018; Thomson et al., 1980).

ASFV is the only member of the Asfarviridae family. Prior studies showed that the ASFV virion has a complex structure with multiple membrane and protein layers (Revilla et al., 2018; Wang et al., 2019), including a genome-containing nucleoid, a core shell, an inner lipid envelope, an icosahedral protein capsid and an external envelope. The first four layers consist of more than 50 proteins (Alejo et al., 2018; Salas and Andrés, 2013), and an external envelope is gained as ASFV buds pass through the plasma membrane (Andrés et al., 2001).

Currently, antigen detection of ASFV includes molecular diagnostic methods (e.g., PCR CRISPR-based detection) and immunoassays. On-site antibody detection has the advantage of being able to detect subclinical infections with high sensitivity, and such strategies represent an important complement to antigen detection. For in-field ASFV antibody detection, enzyme-linked immunosorbent assay (ELISA) is a recommended analysis method by the World Organization for Animal Health (OIE). However, ELISA requires complex laboratory operations and particular equipment. Hence, a rapid and easily performed method is required to facilitate the on-site diagnosis of ASFV.

The lateral flow assay is an ideal technique for one-step ASFV antibody detection due to its great convenience. When developing a lateral flow assay, two important factors that determine the assay’s sensitivity and accuracy must be considered: label material selection and antigen quality.

The gold nanoparticle (AuNPs) is a widely used low-cost label material that enables visual detection in lateral flow assays. However, the use of colloidal gold is largely limited by its low sensitivity. Several novel fluorescence materials with improved sensitivity and accuracy have been introduced for antigen labeling. However, the results of these fluorescence lateral flow assays cannot be read by the naked eye and require specific devices to interpret the results. Here, we optimized the method of preparing AuNPs and produced ultrauniform AuNPs with a diameter of 40 nm. These AuNPs increased the sensitivity and enabled the visual detection of ASFV antibodies.

**FIGURE 1:**
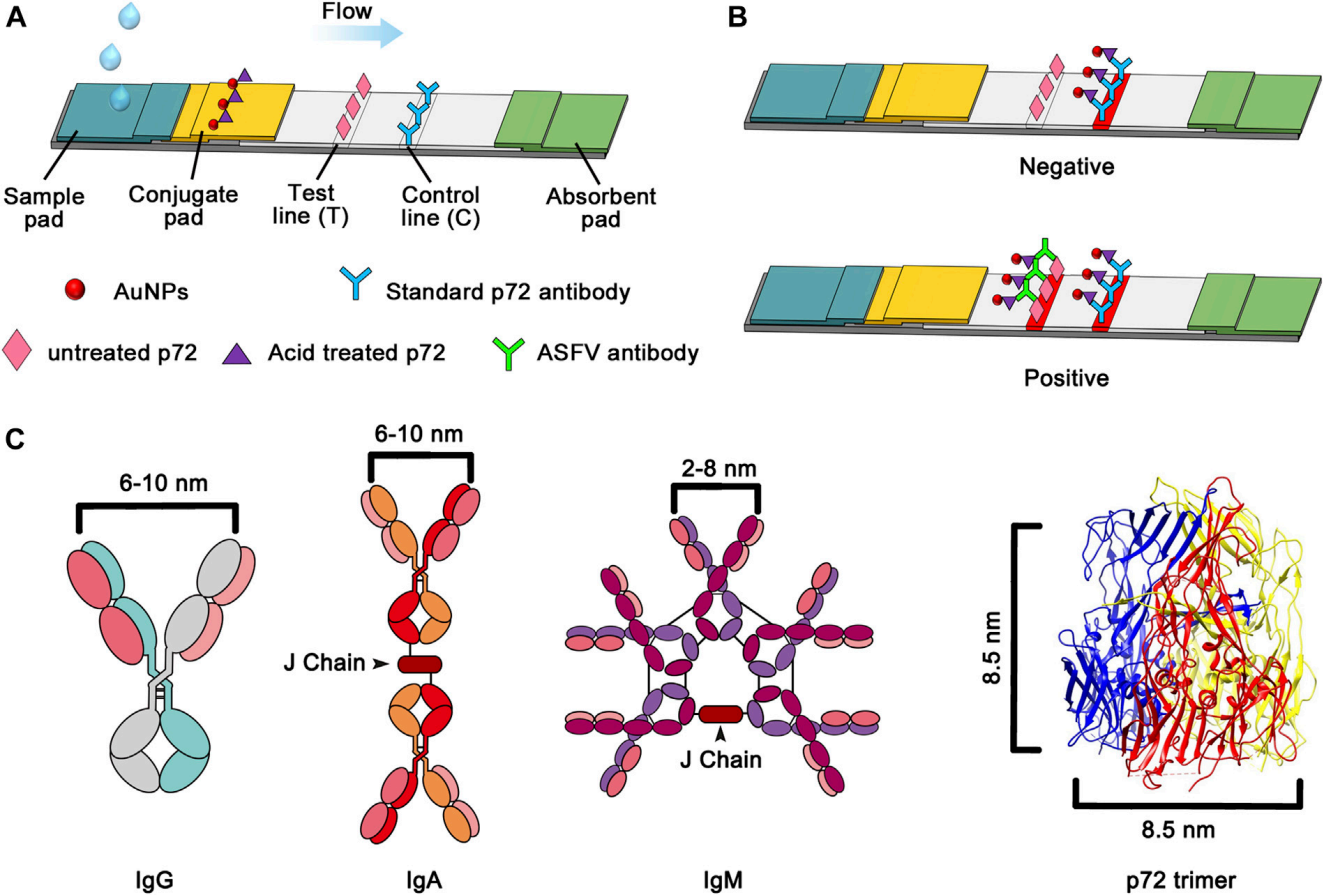
**(A)** Schematic of the proposed sandwich lateral flow strip for ASFV antibody detection. **(B)** Image of the lateral flow assay for positive and negative samples **(C)** Schematic illustration of the distances between the two Fab domains within immunoglobulin and the diameter of the p72 trimer.

The major capsid protein p72, one of the most conserved antigens according to genomic and proteomic analyses (Zhu and Meng, 2020), forms homotrimers and assembles the icosahedral shell of ASFV (Liu et al., 2019; Wang et al., 2019). As p72 accounts for 33% of the total mass of the virus particle (Carrascosa et al., 1984; García-Escudero et al., 1998), it represents a promising capture antigen for the development of an antibody detection method. Liu, Q., et al.(Liu et al., 2019) successfully produced full-length, correctly folded p72. These results pave the way for using full-length p72 in serological diagnostics. In order to increase the sensitivity, double antigen sandwhich lateral flow assay is required with the advantage of antibody detection regardless of isotypes. However, the physical size of p72 trimer is about 8.5 nm in diameter. It may produce steric hindrance for the antibody to bind two identical particles (Figure 1C). The capsid of ASFV, which is assembled with the majority of p72, undergoes a decoating process in low pH during endocytosis (Cuesta-Geijo et al., 2012; Hernáez et al., 2016; Sanchez et al., 2017). It suggests a conformational change of p72 trimer in low pH.

In this article, a rapid detection method for ASFV antibodies was developed using a well-folded p72 trimer and acid-treated p72 prepared by our group as the capture antigen and labeled antigen, respectively. This AuNP-lateral flow assay further combines the advantages of the immune lateral-flow system with the unique properties of AuNPs. The whole test process is completed in less than 15 min to obtain results and requires only 10–20 μL serum. The sensitivity, specificity, and stability were evaluated by testing clinically positive and negative serum samples, and ELISA test results were used as the control. The developed rapid detection method may have great potential for addressing ASFV.

## Materials and Methods

### Reagents and Materials

Ultrauniform AuNPs 40 nm in diameter were fabricated by our lab as previously described (Zhu et al., 2019). In short, primary seed crystals with a size of 15 nm were prepared by the chemical reduction method, and then ultrauniform AuNPs with a size of 40 nm were prepared by the seeding method.

AuNPs with a size of 40 nm were also prepared by traditional methods. One hundred milliliters of 0.01% HAuCl_4_ solution was heated to boiling. Then, 4 ml of 1% sodium citrate solution was added while stirring. The solution turns blue quickly, and heating is continued until the solution turns red.

Bull serum albumin (BSA) was purchased from Sigma-Aldrich (St. Louis, MO, United States). Clinical specimens, including serum from clinically confirmed ASFV-positive pigs and healthy pigs (negative), were provided by Harbin Veterinary Research Institute, Chinese Academy of Agricultural Sciences. Positive sera against pathogenic porcine circovirus (PCV), classical swine fever virus (CSFV), porcine reproductive and respiratory syndrome virus (PRRSV) and pseudorabies virus (PrV) were provided by Harbin Veterinary Research Institute, Chinese Academy of Agricultural Sciences. Sample pads, conjugate pads, nitrocellulose (NC) membranes, absorbent pads and plastic shells were obtained from Shanghai Kinbio Tech. Co., Ltd.

### Preparation of the Capture Antigen and Labeled Antigen

Well-folded p72 trimers were prepared as the capture antigen. The constructed expression cassette carrying the gene encoding p72 was integrated into yeast competent cells. Transformants were selected on plates containing SCD medium. The new strain was grown in YPD at 30°C until the O.D. 600 reached 2.5 to 3.5. Chromatography binding buffer (prepared according to IBA’s manual for Strep-Tactin XT, pH 8.5) was used to resuspend the precipitate after centrifugation, and the product was homogenized. The supernatant of the centrifuged cell lysate was loaded onto a Strep-Tactin XT gravity-flow column (IBA), and affinity chromatography was performed as described in the manufacturer’s manual. The collected eluents were analyzed with SDS–PAGE, and the final pool was selected based on purity.

Deformed p72 was obtained by treatment with a low pH solution. Both well-folded p72 trimers and acid-treated p72 were tested as capture antigen and labeled antigens. To prepare acid-treated p72, p72 trimer samples were then prepared at pH 3 (150 mM NaCl, 100 mM Bis-tris) and incubated for 2 min at 37°C. Size exclusion chromatography (SEC) was employed to verify the uniformity and polymerization state of the untreated and acid-treated p72. The peak fractions were analyzed by 10% SDS–PAGE.

### Preparation of AuNP-p72 Conjugates

Both untreated and acid-treated p72 were conjugated with AuNPs to determine the optimum labeled antigen.

Gold conjugates were synthesized using AuNPs and untreated p72 or acid-treated p72. Briefly, 10 ml of AuNP solution was prepared, p72 protein was added, and the mixture was incubated with gentle shaking at room temperature for 1 h. Then, 1% (w/v) BSA was added to the mixture and shaken constantly for 1 h. After centrifugation of the mixture two times at 10,000 rpm for 30 min to remove the excess BSA, the pellet was resuspended in 10 mM PBS and stored at 4°C.

### Preparation of the Lateral Flow Test Strip

The test strip was composed of a sample pad, conjugate pad, nitrocellulose (NC) membrane and absorbent pad. The conjugation pad was prepared by dispensing a desired volume of AuNP-labeled p72 antigen (AuNP-p72) onto the glass fiber pad using an XYZ Platform Dispenser, followed by drying at 37 °C for 1 h and then storage at 4 °C. Both untreated and acid-treated p72 were sprayed on the test line of the NC membrane to determine the optimum labeled antigen, and a standard p72 antibody was sprayed on the control line. The sample pad, conjugate pad, NC membrane and absorbent pad were sequentially attached to a PVC backing card with a 1–2 mm overlap. The card was then cut into 4 mm wide strips and assembled into a plastic shell for future use.

### Sensitivity and Specificity of the Lateral Flow Test Strip

The sensitivity of the assay signifies the lowest concentration of analyte that can be detected by the developed assay. It is defined here in terms of different dilution rates (1:10, 1:100, 1:1,000, and 1:10,000).

In addition to sensitivity, specificity is another important parameter for a new assay with potential applications. Positive serum samples of PCV, CSFV, PRRSV and PrV were employed to study the specificity of the established lateral flow test strip.

### Validation and Analysis With ELISA Kits

To further validate the lateral flow test strip, eighty-nine clinical serum samples were assessed by using both lateral flow assay and ELISA. The ELISA test result was cross validated by commercial ELISA kits manufactured by Harbin Weike Biotechnology Co., Ltd. and ID vet.

### Animal Experiments

The seven-week-old SPF Large White pig used in this study was supplied by the Laboratory Animal Center of Harbin Veterinary Research Institute. The pig was confirmed to be free of PCV, CSFV, PRRSV, and PrV. Then, the pig was intramuscularly inoculated with a 10^3^ TCID_50_ dose of ASFV attenuated strain (HLJ/HRB1/20) in the biosafety level 3 (BSL3) animal facilities at Harbin Veterinary Research Institute. Blood samples were collected from the jugular vein or anterior vena for antibody detection at the indicated time points, including 0, 3, 5, 7 and 9 days post-infection (DPI). Then separated serum was used to test the ASFV antibody. The experiment was approved by the Laboratory Animal Welfare Committee of Harbin Veterinary Research Institute. All animal-related research procedures complied with the Animal Welfare Act and the Guide for the Care and Use of Laboratory Animals. The virus is prepared as previously prepared (Zhao et al., 2019; Sun et al., 2021).

## Results

### Development of Lateral Flow Test Strip

The lateral flow assay was established into a sandwich structure based on the antibody-antigen reaction showing in Figure 1.

For detection, centrifuge the blood sample for at least 5 min at 4,000 rpm, then mixing 10 µL of a serum with 90 µL of PBS makes a 1:10 dilution. The 100 μL of the mixture was added to the sample port of the test strip, wait for 10 min for test result interpretation. The liquid flowed toward the absorbent pad under the hydrodynamic force and capillary power. Antibodies in the serum were captured by the probe AuNP conjugate complex. Then, the probe-antibody compound was transferred to the NC membrane. The well-folded p72 trimer immobilized at the test line (T) caught the target antibody against ASFV and formed a characteristic red band due to the formation of the probe-antibody-p72 trimer compound. In addition, once the sample passed through the control line (C), the immobilized standard p72 antibody directly caught the excess AuNP conjugate probe; thus, a second red band appeared on the C line. In the absence of the target antibody, the red band can only be observed on the C line. Based on this response principle, the ASFV antibody analysis can be simply performed by observing the color change of the T line with the naked eye. If there was no visible C line, whether a red T line appeared or not, the test result was judged as invalid. Semi-quantitative analysis of antibodies could be achieved by determining color intensity (measured in gray value) of the T line and C line, and calculating T/C.

### Optimization of Lateral Flow Test Strip

To improve the performance of the lateral flow assay, factors including the AuNP radius and the state of p72 in the AuNP conjugate complex were explored.

**FIGURE 2:**
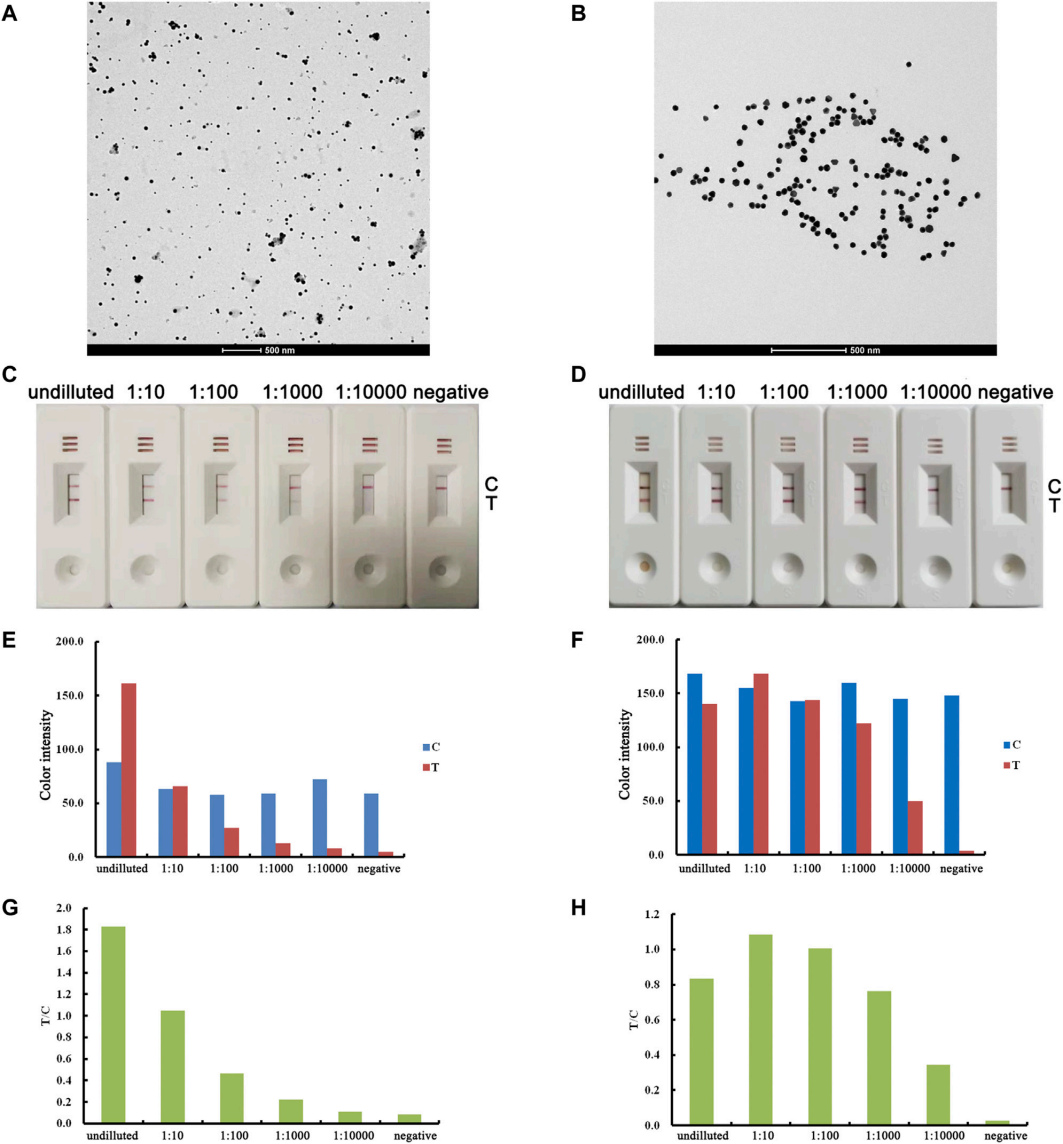
Optimization of the AuNP label. The results for traditional AuNPs are shown in the left column, and the results for ultrauniform AuNPs are shown in the right column. TEM images of AuNPs **(A, B)**. Real detection figures of sensitivity analysis of ASFV antibody by lateral flow assay **(C, D)**. The color intensity values of the test line (T value) and control line (C value) of the lateral flow assay **(E, F)**. The T/C value of lateral flow assay **(G, H)**.

To improve the sensitivity of AuNPs, we fabricated ultrauniform AuNPs (40 nm) and carried out a comparison study between the ultrauniform AuNPs and AuNPs fabricated by the traditional method. The prepared AuNPs were characterized by TEM before conjugation. As a result, we observed that traditional AuNPs present an uneven shape and aggregated particles (Figure 2A), while ultrauniform AuNPs present an average diameter of 40 nm, are all spherical in shape and possess a uniform size distribution (Figure 2B). The prepared AuNPs were then applied for lateral flow assay development. For the positive sample, the T line remained visible to the 1:10,000 diluted sample when using ultrauniform AuNPs as labels (Figure 2D, F, H). Meanwhile, the limit of detection for the traditional AuNPs was 1:1,000 (Figures 2C, E, G). The results indicated that ultrauniform AuNPs are more suitable than traditional AuNPs as a label.

**FIGURE 3:**
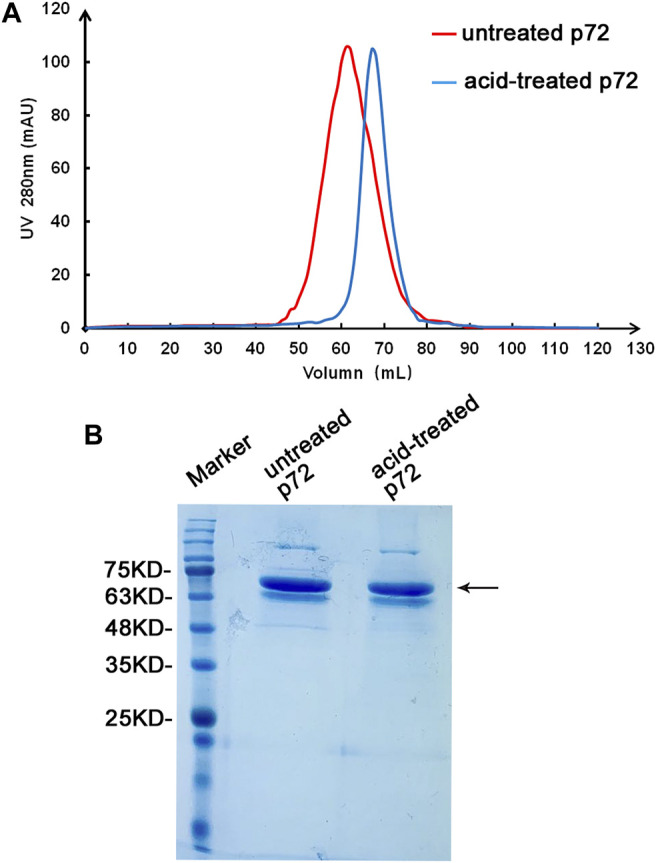
Preparation of the p72 antigen. Analysis of untreated and acid-treated p72 by SEC **(A)**. The band corresponding to p72 on the stained SDS-PAGE gel **(B)** is indicated by an arrow.

**FIGURE 4:**
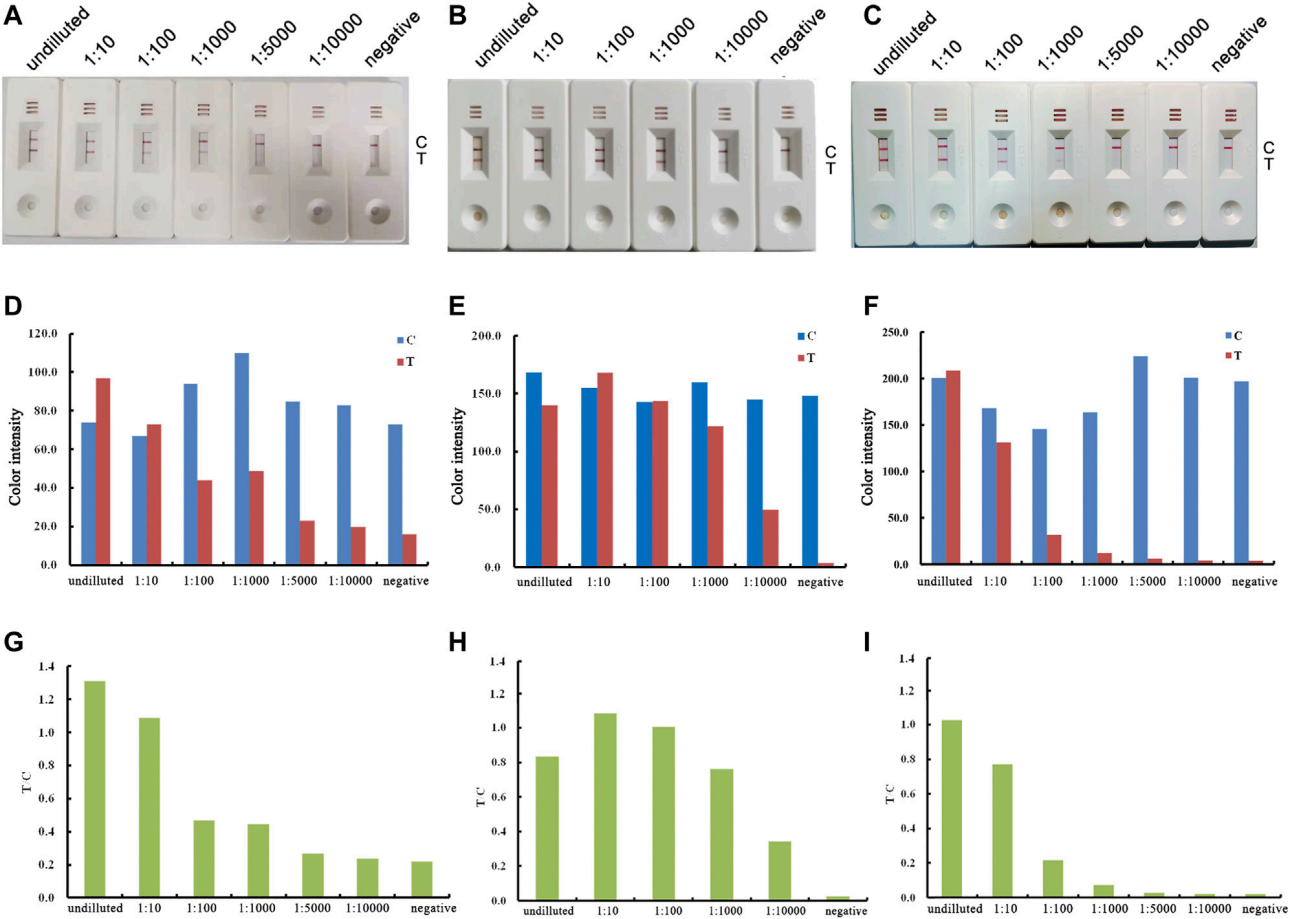
Optimization of the labeled antigen and capture antigen. The results of using untreated p72 trimer as both labeled antigen and capture antigen are shown in the left column **(A, D, G)**; the results of using acid-treated p72 as labeled antigen, untreated p72 trimer as capture antigen are shown in the middle column **(B, E, H)**; the results of using acid-treated p72 as both labeled antigen and capture antigen are shown in the right column **(C, F, I)**. Real detection figures of sensitivity analysis of ASFV antibody by lateral flow assay **(A, B, C)**. The color intensity values of the test line (T value) and control line (C value) of the lateral flow assay **(D, E, F).** The T/C value of lateral flow assay **(G, H, I)**.

To determine the optimum state of p72 when preparing the labeled antigen, both untreated and acid-treated p72 were conjugated with AuNPs. Both untreated and acid-treated p72 were subsequently purified by size exclusion chromatography (SEC). The results showed that after the acid treatment, the eluting peak of p72 shifted backward (Figure 3A). The peaks of untreated and treated p72 corresponded to ∼210 and 100 kDa, respectively, indicating that the p72 trimers may have undergone deformation after acid treatment. The peak fractions were gathered and analyzed by SDS–PAGE (Figure 3B). After conjugation with AuNPs, the conjugation complex solution was stable and aggregation was not observed. By adopting a series concentration of positive serum, the sensitivity of strips using untreated p72 or acid-treated p72 as labeled antigen was determined by the naked eye. For strips using acid-treated p72 as the labeled antigen, the color of the T line remained even when the positive sample was diluted 1:10,000 (Figures 4B,E,H). However, the same line disappeared at a dilution of 1:5,000 in the assay using the well-folded p72 trimer as the labeled antigen (Figures 4A,D,G). This may be due to the steric hindrance provided by the p72 trimer when an antibody-p72 trimer complex formed, which would hinder the interaction of another p72 trimer with the complex (Figure 1C). Previous research has revealed that the Fab-Fab distances within IgG, IgM and IgA are 6–10 nm, 2–8 nm and 6–10 nm, respectively (Loset et al., 2004; Samsudin et al., 2020; Su et al., 2018; Werner et al., 1972; Zhang et al., 2020; Zhang et al., 2015), while the particle size of the p72 trimer is 8.5 nm × 8.5 nm (Liu et al., 2019). Thus, by treating p72 with acid, the size of the antigen decreased, which facilitated its interaction with the antibody-p72 trimer complex. We also examine the quality of test strip when using acid-treated p72 as both capture antigen and labeled antigen. Unfortunately, the test line almost disappeared at a dilution of 1:1,000 in the assay (Figures 4C,F,I).

### Sensitivity of the Lateral Flow Test Strip

The sensitivity of the optimized test strip maintains the same performance as the test strip prepared by the new method in the optimization experiment. The positive serum sample was selected based on the cross validation result by commercial ELISA kits (Harbin Weike Biotechnology Co. Ltd., China and ID vet, France). Positive serum samples were diluted to different concentrations and added to the strip. After diluting the positive serum sample to four dilution factors (1:10, 1:100, 1:1,000 and 1:10,000) with distilled water, six lateral flow strips were tested (with one undiluted sample and one negative control strip). The limit of detection was determined by the visibility of test lines on the strips. When the diluted positive serum sample was tested, the color intensity of the T line slowly diminished as the antibody level decreased (Figure 4B, from left to right). When the dilution ratio reached 1:10,000, the red T line could still be observed by the naked eye. Hence, a dilution of 1:10,000 was chosen as the limit of detection for the lateral flow strip.

### Specificity of the Lateral Flow Test Strip

**FIGURE 5:**
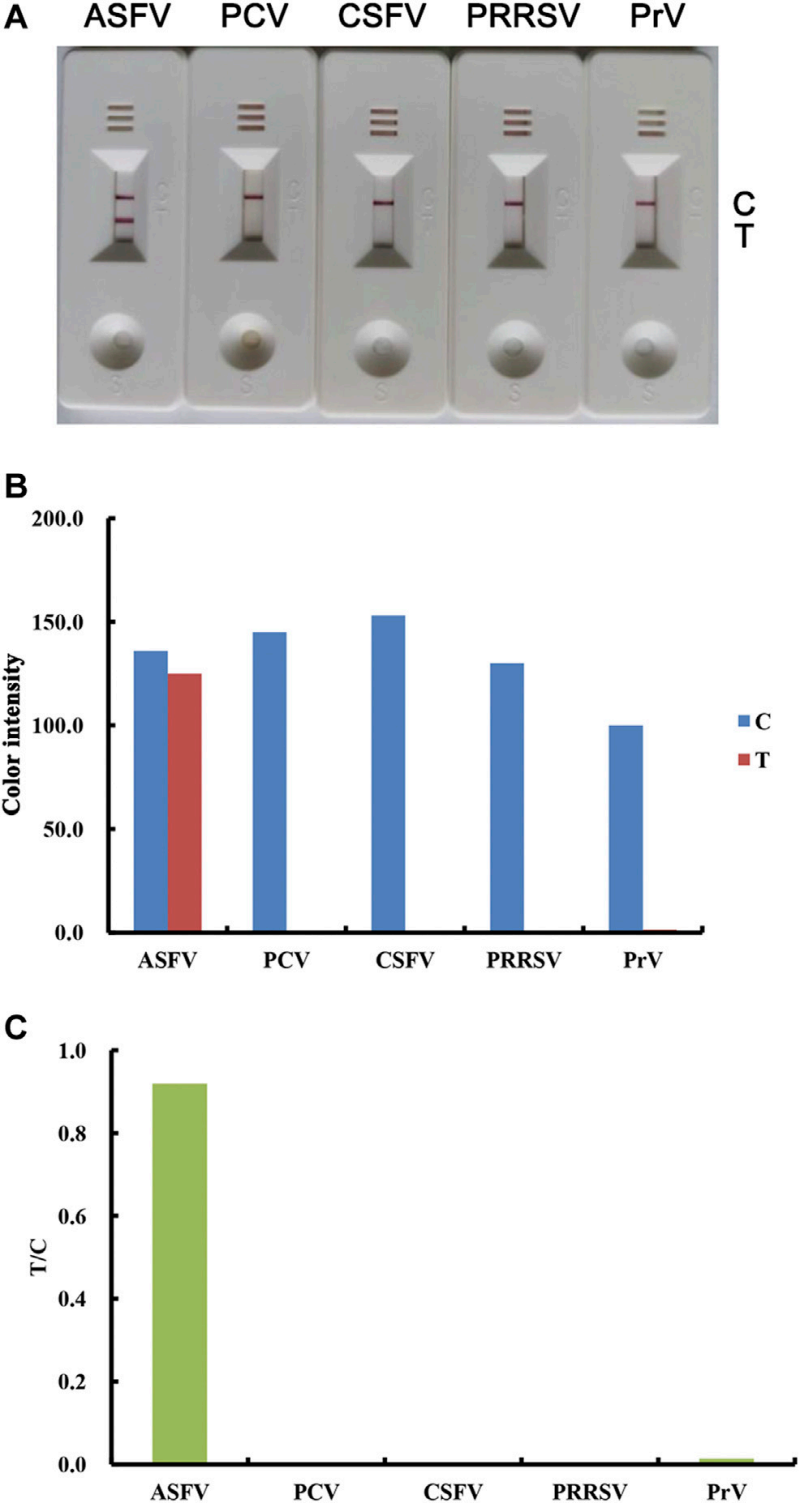
Specificity of the lateral flow strip for the detection of ASFV antibodies. Real detection figures of sensitivity analysis of ASFV antibody by lateral flow assay **(A)**. The color intensity values of the test line (T value) and control line (C value) of the lateral flow assay **(B)**. The T/C value of the lateral flow assay **(C)**.

Specificity is an important parameter for a new strip in preventing false-positive results from cross-reaction. To determine the specificity of the lateral flow strip toward the ASFV antibody, clinical serum samples against ASFV, PCV, CSFV, PRRSV and PrV were tested. As shown in Figure 5, only ASFV-positive serum samples produced a legible red band on the T line, while other samples showed no visible T lines. These results demonstrated that there was no cross-reactivity between ASFV and the other listed viruses.

### Reproducibility of the Lateral Flow Test Strip

To determine the reproducibility of the results of the lateral flow test strip, five replicates were prepared and tested (Supplementary Figure S1). The coefficient of variation (CV) of T line color intensity and T/C value were found to be 1.7 and 2.2%, respectively. This result demonstrated that our developed lateral flow strip possesses great reproducibility and potential for use in commercial applications.

### Detection of Clinical Samples by the Lateral Flow Test Strip

The final purpose of the work was to establish a rapid and reliable method in which the antibody against ASFV could be detected directly by the naked eye. To evaluate the prepared strip’s practicality, 89 clinical serum samples were collected and tested. A standard commercial ELISA kit was utilized for comparison. The experimental results showed that the response of the strips to clinical serum samples were consistent with the cross validation result by commercial ELISA kits (Table 1). These results demonstrated that this strip assay has good reliability and can be applied to the practical application of rapid and on-site surveillance of ASFV infection.

**TABLE 1:** Results of the lateral flow assay and commercial ELISA.

Assay	Detecting rate		Coincidence rate		Overall coincidence rate
	Positive	Negative	Positive	Negative	
Lateral flow test strip	61.8% (55/89)	38.2% (34/89)	98% (54/55)	97% (33/34)	97.8% (87/89)
commercial ELISA kit	61.8% (55/89)	38.2% (34/89)			

Serum antibody responses to ASFV inoculation by the lateral flow test strip.

**FIGURE 6:**
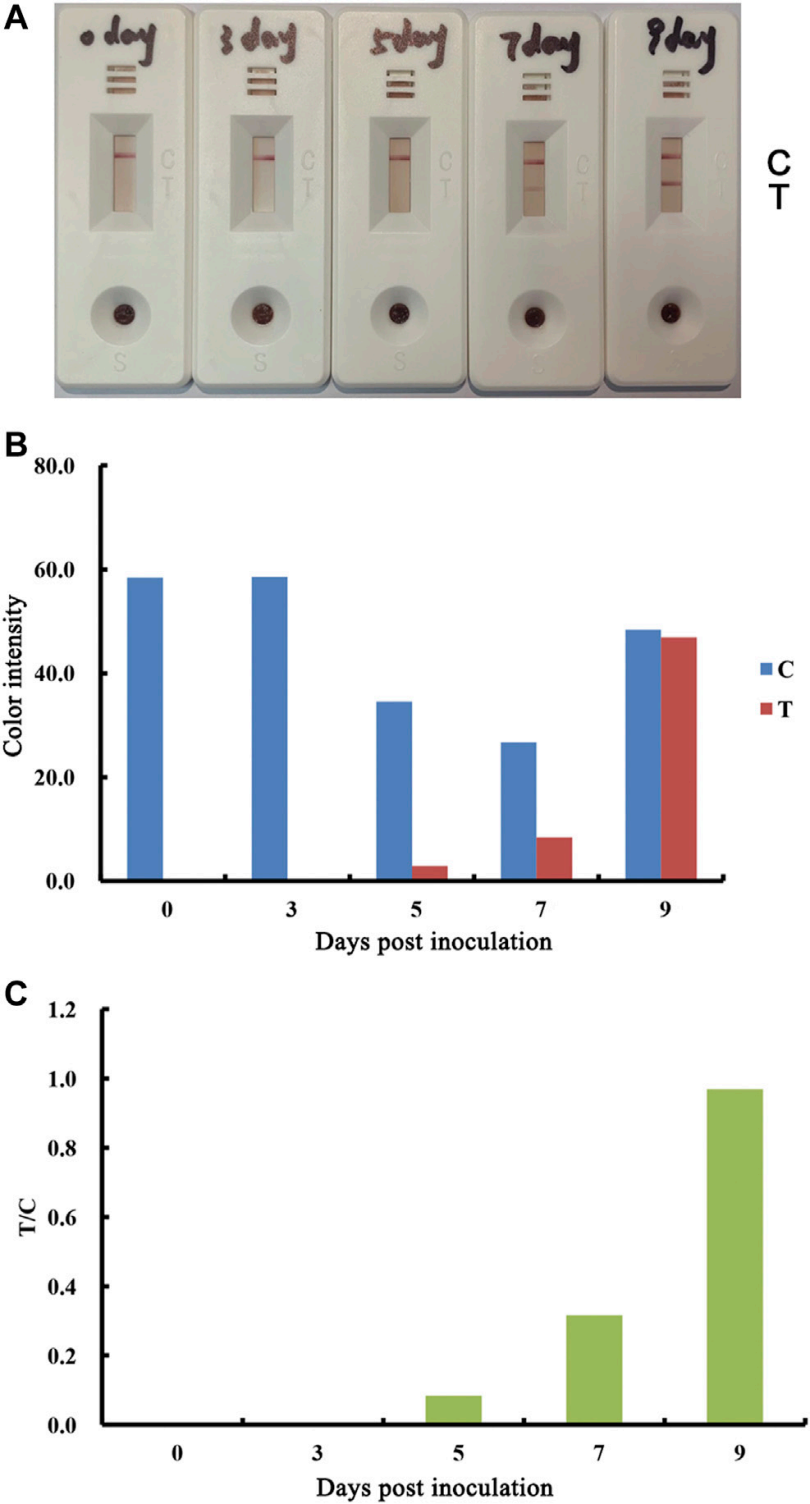
Performance of lateral flow strip for the detection of ASFV antibodies in early stage of exposure. Real detection figures of ASFV antibodies by lateral flow assay. Blood samples were collected at the indicated timepoints from pigs inoculated with 10^3^ doses of ASFV **(A)**. The color intensity values of the test line (T value) and control line (C value) of the lateral flow assay **(B)**. The T/C value of the lateral flow assay **(C)**.

The test strip was also evaluated to determine whether it is suitable for the early stage post exposure. Blood samples collected between 0 and 9 days post-infection (DPI) with ASFV were tested for serum antibodies. The results showed that antibody responses were observed at DPI≥7 (Figure 6). In addition, the antibody responses at DPI seven were significantly greater than the responses at DPI 9.

## Conclusion

This study successfully established a lateral flow test strip to detect ASFV antibodies in clinical serum samples. The testing results can be obtained in less than 15 min without the use of any additional devices. We also provided an antibody semi-quantitative analysis method. Our results demonstrate that the lateral flow strip has high specificity for ASFV antibodies and good sensitivity for the detection of clinical serum samples diluted by 1:10,000. The established strip showed high positive and negative coincidence rates compared to the commercial ELISA kits, which were 98 and 97%, respectively. The serum antibody response evaluation to ASFV inoculation showed that the established strip could be applied for early-stage serological diagnostics. The lateral flow strip can also be widely employed to help epidemiological investigations of ASFV.

Of note, the lateral flow strip surpassed commercial ELISA kits in terms of cost effectiveness, stability, reaction time and ease of use (Supplementary Table S1). To date, the lateral flow test strips based on the dual quantum dot (QDM) microsphere, latex microsphere, or fluorescent microsphere were also developed for ASFV antibodies detection (Sastre et al., 2016; Li et al., 2020; Li et al., 2022) (Supplementary Table S1). Compared with these methods, the AuNP based test strip developed in this study, has the advantages of no instrument requirement, shorter time consumption, and lower cost. However, our lateral flow strip is a qualitative and semi-quantitative method, cannot accurately quantify the content of ASFV antibody similar to QMD based test strip. Given high requirements in the analytical field, it is necessary to optimize the accuracy of this semi-quantitative method by studying the relationship between antibody concentration and the T line color intensity.

## Data Availability

The original contributions presented in the study are included in the article/Supplementary Material, further inquiries can be directed to the corresponding authors.

## References

[B1] Alejo A. Matamoros T. Guerra M. Andrés G. (2018). A Proteomic Atlas of the African Swine Fever Virus Particle. J. Virol. 92, e01293-18. 10.1128/JVI.01293-18 30185597 PMC6232493

[B2] Anderson E. C. Hutchings G. H. Mukarati N. Wilkinson P. J. (1998). African Swine Fever Virus Infection of the Bushpig (*Potamochoerus porcus*) and its Significance in the Epidemiology of the Disease. Vet. Microbiol. 62, 1–15. 10.1016/s0378-1135(98)00187-4 9659687

[B3] Andrés G. García-Escudero R. Viñuela E. Salas M. a. L. Rodríguez, J. M. (2001). African Swine Fever Virus Structural Protein pE120R Is Essential for Virus Transport from Assembly Sites to Plasma Membrane but Not for Infectivity. J. Virol. 75, 6758. 11435554 10.1128/JVI.75.15.6758-6768.2001 PMC114402

[B4] Carrascosa J. Carazo J. Carrascosa A. L. García N. Santisteban A. Viñuela E. (1984). General Morphology and Capsid fine Structure of African Swine Fever Virus Particles. Virology 132, 160–172. 10.1016/0042-6822(84)90100-4 6695498

[B5] Cuesta-Geijo M. A. Galindo I. Hernáez B. Quetglas J. I. Dalmau-Mena I. Alonso C. (2012). Endosomal Maturation, Rab7 GTPase and Phosphoinositides in African Swine Fever Virus Entry. PLOS ONE 7, e48853. 10.1371/journal.pone.0048853 23133661 PMC3486801

[B6] García-Escudero R. Andrés G. Almazán F. Viñuela E. (1998). Inducible Gene Expression from African Swine Fever Virus Recombinants: Analysis of the Major Capsid Protein P72. J. Virol. 72, 3185–3195. 9580160 10.1128/jvi.72.4.3185-3195.1998 PMC109780

[B7] Hernáez B. Guerra M. Salas M. L. Andrés G. (2016). African Swine Fever Virus Undergoes Outer Envelope Disruption, Capsid Disassembly and Inner Envelope Fusion before Core Release from Multivesicular Endosomes. Plos Pathog. 12, e1005595. 27110717 10.1371/journal.ppat.1005595 PMC4844166

[B8] Li C. He X. Yang Y. Gong W. Huang K. Zhang Y. (2020). Rapid and Visual Detection of African Swine Fever Virus Antibody by Using Fluorescent Immunochromatography Test Strip. Talanta 219, 121284. 10.1016/j.talanta.2020.121284 32887174

[B9] Li J. Bai Y. Li F. Zhang Y. Xie Q. Zhang L. (2022). Rapid and Ultra-sensitive Detection of African Swine Fever Virus Antibody on Site Using QDM Based-ASFV Immunosensor (QAIS). Analytica Chim. Acta 1189, 339187. 10.1016/j.aca.2021.339187 34815032

[B10] Liu Q. Ma B. Qian N. Zhang F. Tan X. Lei J. (2019). Structure of the African Swine Fever Virus Major Capsid Protein P72. Cell Res 29, 953–955. 10.1038/s41422-019-0232-x 31530894 PMC6889146

[B11] Løset G. Å. Roux K. H. Zhu P. Michaelsen T. E. Sandlie I. (2004). Differential Segmental Flexibility and Reach Dictate the Antigen Binding Mode of Chimeric IgD and IgM: Implications for the Function of the B Cell Receptor. J. Immunol. 172, 2925–2934. 10.4049/jimmunol.172.5.2925 14978095

[B12] Parker J. Plowright W. Pierce M. A. (1969). The Epizootiology of African Swine Fever in Africa. Vet. Rec. 85, 668–674. 5391024

[B13] Revilla Y. Pérez-Núñez D. Richt J. A. (2018). African Swine Fever Virus Biology and Vaccine Approaches. Adv. Virus. Res. 100, 41–74. 10.1016/bs.aivir.2017.10.002 29551143

[B14] Salas M. L. Andrés G. (2013). African Swine Fever Virus Morphogenesis. Virus. Res. 173, 29–41. 10.1016/j.virusres.2012.09.016 23059353

[B15] Samsudin F. Yeo J. Y. Gan S. K.-E. Bond P. J. (2020). Not all Therapeutic Antibody Isotypes Are Equal: the Case of IgM versus IgG in Pertuzumab and Trastuzumab. Chem. Sci. 11, 2843–2854. 10.1039/c9sc04722k 32206268 PMC7069520

[B16] Sanchez E. G. Perez-Nunez D. Revilla Y. (2017). Mechanisms of Entry and Endosomal Pathway of African Swine Fever Virus. Vaccines 5, 42. 10.3390/vaccines5040042 PMC5748609 29117102

[B17] Sánchez-Cordón P. J. Montoya M. Reis A. L. Dixon L. K. (2018). African Swine Fever: A Re-emerging Viral Disease Threatening the Global Pig Industry. Vet. J. 233, 41–48. 10.1016/j.tvjl.2017.12.025 29486878 PMC5844645

[B18] Sastre P. Pérez T. Costa S. Yang X. Räber A. Blome S. (2016). Development of a Duplex Lateral Flow Assay for Simultaneous Detection of Antibodies against African and Classical Swine Fever Viruses. J. Vet. Diagn. Invest. 28, 543–549. 10.1177/1040638716654942 27400954

[B19] Su C. Lua W.-H. Ling W.-L. Gan S. (2018). Allosteric Effects between the Antibody Constant and Variable Regions: A Study of IgA Fc Mutations on Antigen Binding. Antibodies 7, 20. 10.3390/antib7020020 PMC6698812 31544872

[B28] Sun E. Zhang Z. Wang Z. He X. Zhang X. Wang L. (2021). Emergence and Prevalence of Naturally Occurring Lower Virulent African Swine Fever Viruses in Domestic Pigs in China in 2020. Sci. China Life Sci. 64 (5), 752–765. 10.1007/s11427-021-1904-4 33655434

[B20] Thomson G. R. Gainaru M. D. Van Dellen A. F. (1980). Experimental Infection of Warthos (Phacochoerus Aethiopicus) with African Swine Fever Virus. Onderstepoort J. Vet. Res. 47, 19–22. 7454231

[B21] Wang N. Zhao D. Wang J. Zhang Y. Wang M. Gao Y. (2019). Architecture of African Swine Fever Virus and Implications for Viral Assembly. Science 366, 640–644. 10.1126/science.aaz1439 31624094

[B22] Werner T. C. Bunting J. R. Cathou R. E. (1972). The Shape of Immunoglobulin G Molecules in Solution. Proc. Natl. Acad. Sci. 69, 795–799. 10.1073/pnas.69.4.795 4502932 PMC426566

[B23] Zhang P. Liu X. Liu P. Wang F. Ariyama H. Ando T. (2020). Capturing Transient Antibody Conformations with DNA Origami Epitopes. Nat. Commun. 11, 3114. 10.1038/s41467-020-16949-4 32561744 PMC7305102

[B24] Zhang X. Zhang L. Tong H. Peng B. Rames M. J. Zhang S. (2015). 3D Structural Fluctuation of IgG1 Antibody Revealed by Individual Particle Electron Tomography. Sci. Rep. 5, 9803. 10.1038/srep09803 25940394 PMC4419541

[B29] Zhao D. Liu R. Zhang X. Li F. Wang J. Zhang J. (2019). Replication and virulence in pigs of the first African swine fever virus isolated in China. Emerg Microbes Infect, 8(1), 438–447. 10.1080/22221751.2019.1590128 30898043 PMC6455124

[B25] Zhou X. Li N. Luo Y. Liu Y. Miao F. Chen T. (2018). Emergence of African Swine Fever in China, 2018. Transbound Emerg. Dis. 65, 1482–1484. 10.1111/tbed.12989 30102848

[B26] Zhu W. Liu Y. Meng G. (2019). Preparation and Characterization of Nano-Gold Particles by Seeding Method. Chin. J. Vet. Med. 55, 33–35+130. (in Chinese).

[B27] Zhu Z. Meng G. (2020). ASFVdb: an Integrative Resource for Genomic and Proteomic Analyses of African Swine Fever Virus. Database (Oxford) 2020, baaa023. 10.1093/database/baaa023 32294195 PMC7159030

