# Supplementary material for: Lateral Flow Assay for the Detection of African Swine Fever Virus Antibodies Using Gold Nanoparticle-Labeled Acid-Treated p72
Source: Front Chem. 2022 Jan 3;9:804981. doi: 10.3389/fchem.2021.804981 (PMC8761911; doi:10.3389/fchem.2021.804981)
Supplement: Supplementary file 1 [file DataSheet1.docx]

Supplementary Material


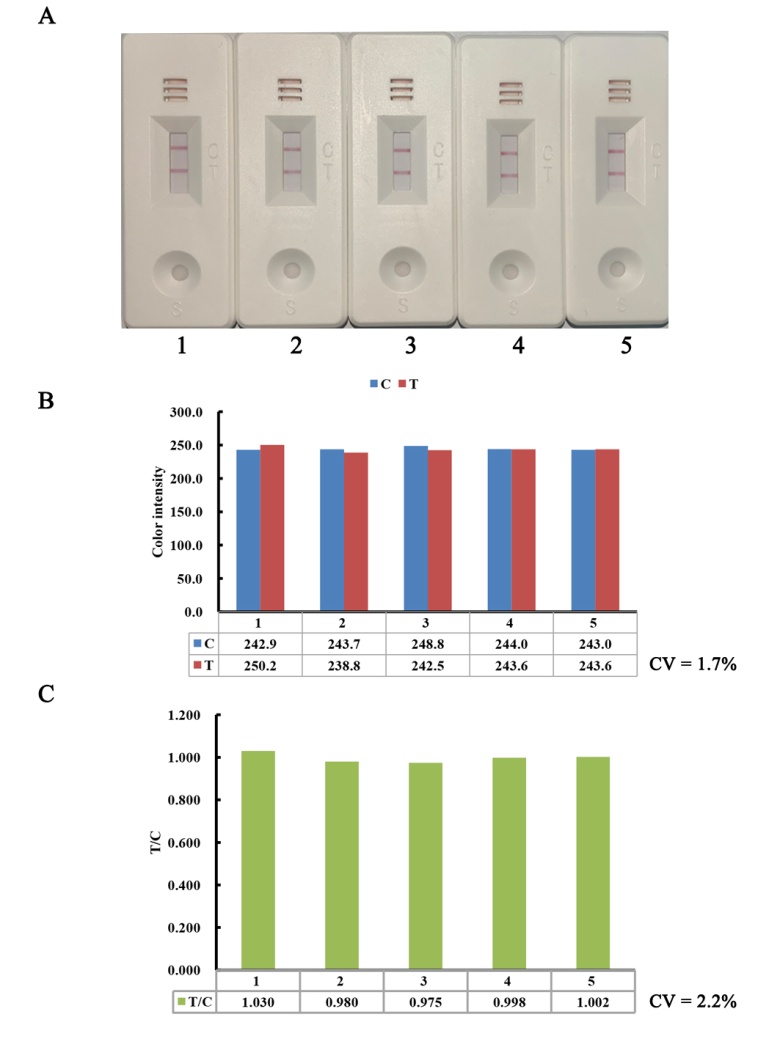


**Figure S1. Reproducibility of the lateral flow test strip.** Real detection figures of the lateral flow assay repeat five times on the same sample (A). The color intensity values of the test line (T value) and control line (C value) of the lateral flow assay (B). The T/C value of the lateral flow assay (C).

**Table S1. Comparison of methods on ASFV antibody serological detection**

| **Detection method** | **Antigen** | **Qualitative or quantitative** | **Time consumption** | **Sensitivity** | **Instrument requirment** | **Reference** |
| --- | --- | --- | --- | --- | --- | --- |
| Lateral flow test strip established by this method | p72 | Qualitative and semi-quantitative | 10 min | 1:10000 | Required no instruments for qualitative testing, but need smartphone application for semi-quantitative |  |
| Dual quantum dot microsphere (QDM) based lateral flow test strip | p30, p54 | Quantitative | 25 min | 1:1000 to 1:64000 | Require handheld fluorescence immunoassay analyzer for result interpretation | (Li et al., 2022) |
| Latex microsphere based lateral flow test strip | p72 | Qualitative and semi-quantitative | 10 min | 1:3200, 0.96 ng/test | Required no instruments for qualitative testing, but need lateral flow assay reader for semi-quantitative | (Sastre et al., 2016) |
| Fluorescent immunochromatography test strip | Truncated p54 | Quantitative | 20 min | 1:640 | Require fluorescent strip reader for result interpretation | (Li et al., 2020) |
| Indirect ELISA | Truncated p72, P62 and P30 (IDvet-ELISA), p54 (Biostone-ElISA), p72 (HARVAC-ELISA) | Quantitative | 1-2 h | * | Require microplate absorbance reader for result interpretation | IDVet, Biostone,  Harbin HARVAC biotechnology |
| Blocking ELISA | Truncated p72 (INGENASA-ELISA), p30 | Quantitative | 2-3 h | NA* | Require microplate absorbance reader for result interpretation | INGENASA,  (Yuan et al., 2021) |

* The detection limit of ASFV antibody ELISA kit is not available. Generally, ELISA shows higher sensitivity than lateral flow assay. However, blocking ELISA has lower sensitivity relative to indirect ELISA.

Reference:

Li, C., He, X., Yang, Y., Gong, W., Huang, K., Zhang, Y., Yang, Y., Sun, X., Ren, W., Zhang, Q.*, et al.* (2020). Rapid and visual detection of African swine fever virus antibody by using fluorescent immunochromatography test strip. Talanta *219*, 121284.

Li, J., Bai, Y., Li, F., Zhang, Y., Xie, Q., Zhang, L., Hua, L., Xiong, Q., Shan, Y., Bu, Z.*, et al.* (2022). Rapid and ultra-sensitive detection of African swine fever virus antibody on site using QDM based-ASFV immunosensor (QAIS). Anal Chim Acta *1189*, 339187.

Sastre, P., Perez, T., Costa, S., Yang, X., Raber, A., Blome, S., Goller, K.V., Gallardo, C., Tapia, I., Garcia, J.*, et al.* (2016). Development of a duplex lateral flow assay for simultaneous detection of antibodies against African and Classical swine fever viruses. J Vet Diagn Invest *28*, 543-549.

Yuan, F., Petrovan, V., Gimenez-Lirola, L.G., Zimmerman, J.J., Rowland, R.R.R., and Fang, Y. (2021). Development of a Blocking Enzyme-Linked Immunosorbent Assay for Detection of Antibodies against African Swine Fever Virus. Pathogens *10*.
